# Triple Encapsulation and Controlled Release of Vancomycin, Rifampicin and Silver from Poly (Methyl Methacrylate) or Poly (Lactic-Co-Glycolic Acid) Nanofibers

**DOI:** 10.3390/bioengineering11060529

**Published:** 2024-05-23

**Authors:** John Jackson

**Affiliations:** Faculty of Pharmaceutical Sciences, University of British Columbia, 2405 Wesbrook Mall, UBC, Vancouver, BC V6T 1Z3, Canada; jackson@mail.ubc.ca

**Keywords:** rifampicin, vancomycin, silver sulfadiazine, polymer nanofibers, controlled release

## Abstract

Although the incidence of infections in orthopedic surgeries, including periprosthetic surgeries, remains low at approximately 1–2%, the number of surgeries and the incidence of drug-resistant bacteria is increasing. The cost and morbidity associated with revision surgeries are huge. More effective drug combinations and delivery methods are urgently needed. In this paper, three anti-infective drugs (vancomycin, rifampicin, and silver sulfadiazine) have been jointly and effectively electrospun in thin (0.1 mm) flexible nanofiber mats of either poly (methyl methacrylate) (PMMA) or poly (lactic-co-glycolic acid) (PLGA). The inclusion of poly (ethylene glycol) (PEG) enabled optimal drug release with a reduced water contact angle for wetting. The controlled release of these three agents from 20% PEG (*w*/*w* to polymer)-blended PMMA or PLGA nanofiber mats may allow for the prophylactical prevention of implant-related infections or provide methods to treat orthopedic infections at the time of revision surgeries. These combinations of drugs provide excellent additive or synergistic antibiotic action against a broader spectrum of bacteria than each drug alone.

## 1. Introduction

With an aging population worldwide, the number of orthopedic surgeries is increasing each year. Infections usually arise from Gram-positive *staphylococcus* (e.g., *S. aureus* at >50% occurrence) but also Gram-positive *streptococcus* and *enterococcus* (approx. 10%) as well as, to a lesser extent, from difficult-to-treat Gram-negative bacteria (<10%) including the *pseudomonas* strain. Periprosthetic joint surgeries are associated with a low but significant (1–2%) incidence of infection (PTI), but the associated morbidity and rehospitalization costs are very high [1]. Revision methods for treating such infections usually involve the surgical removal of the implant, debridement, and the insertion of an antibiotic drug-loaded “spacer” implant made from poly (methyl methacrylate) (PMMA) bone cement for up to six weeks. This temporary implant is then removed, and a repeat operation is performed with variable levels of effectiveness (approximately 85%) [2].

Gentamicin was the first drug used for these purposes, but as bacteria became resistant to the drug, it was replaced by vancomycin, which is a particularly potent antibiotic against Gram-positive bacteria *S. Aureus*, including the resistant methicillin-resistant *S. Aureus* (MRSA) form [3,4]. Because it is estimated that 20% of the common *S. Aurues* is in the MRSA form in hospitals, vancomycin has become a frequently orthopedic drug and is even commonly used prophylactically by sprinkling dry powder into (e.g., spinal surgery sites) wounds with good anti-infective results [5]. The extensive and liberal use of vancomycin has led to concerns about further drug resistance development, and strategies to improve the effectiveness of drugs in spacers, leading to the use of more than one drug [4,6,7]. This has led to improved antibiotic efficacy in two-stage revision (spacer) methods for PTI using vancomycin with either gentamicin [3,4] or rifampicin [6,7]. However, there is increasing concern about high levels of patients (approx. 14%) who develop acute kidney injury resulting in the use of spacers with such high levels of antibiotics [3].

In order to improve the efficacy of drugs like vancomycin, gentamicin, and rifampicin, we and others have investigated the use of additives on bone cement to increase the drug release rate or via the use of silver to allow for improved antibacterial effects, allowing the use of lower drug doses [8,9,10]. Silver has been shown to have additive effects with vancomycin [11,12,13] and rifampicin [14,15] and includes the benefit of expanding the spectrum of bacterial killing to Gram-negative bacteria [11,15]. 

Bone cement (PMMA) is used extensively in orthopedic surgery and is often used to fix prostheses in place in primary replacement surgery. There is an urgent need for antibacterial compositions containing cocktails of drugs that may prevent or treat orthopedic infections, especially from drug-resistant species. The objective of this study was to encapsulate vancomycin, rifampicin, and silver together in an easy-to-handle PMMA film composition that releases three drugs in a controlled manner for a potent three-drug antibacterial effect. Such a film might be wrapped around a prosthesis implant at the time of surgery to prevent bacterial adherence and kill any other introduced bacteria in the surgical area. Alternatively, a film may be used in any open orthopedic surgical site. 

Although polycaprolactone (PCL) [16] and PMMA [17] are strong brittle polymers, they may be rendered flexible if electrospun into nanofibers [16,17]. For PMMA, the inclusion of polyethylene glycol (PEG) reduces the polymer–water contact angle (i.e., increases wettability) and increases drug release rates [17]. Clearly, this polymer is an obvious candidate for implant settings, whereas a biodegradable polymer like PLGA might be more suitable where bone cement is not used. 

## 2. Materials and Methods

Vancomycin, rifampicin, and silver sulfadiazine were obtained from Sigma-Aldrich (St. Louis, MO, USA). PMMA (350 kDa) and PEG were obtained from Sigma-Aldrich (St. Louis, MO, USA). PLGA (50/50 IV = 0.15) was obtained from LACTEL, DURECT Corporation (Birmingham Al). The common notation for PEG is that the number indicates the molecular weight so PEG 1000 has a molecular weight of 1 kDa and PEG 200 k has a molecular weight of 200 kDa. These terms are used interchangeably in the text and figures. 

PMMA with PEG was dissolved in tetrahydrofuran/dimethyl formamide (50% THF/50% DMF) at 15% of the polymer *w*/*v*. The drugs were added at 3% *w*/*w* to the polymer, and the solution was tip-sonicated for 2 min intermittently to avoid heating. This solution was too viscous to spin and was diluted to a 10% solution with 50:50 (THF/DMF). PLGA with or without PEG was dissolved in a similar fashion using a final polymer concentration of 30% in THF/DMF (50:50), and the drugs added to this to a final concentration of 3% *w*/*w* each with tip sonication the same as PMMA. Overall, PEG was blended at 10%, 20%, or 30% (*w*/*w*) concentrations in the PMMA and PLGA polymers. 

The polymers were electrospun using a commercial electrospinning machine (Kato Tech Co., Kyoto, Japan) using 30 kV at a 15 cm range and a flow rate of 0.1 mm/min in a 10 mL syringe. Nanofibers were collected on aluminum foil and stored in bags in a fridge after full drying. 

### 2.1. Drug Release 

Film sections (53 mg) were placed in 10 mL of 10 mM phosphate-buffered saline (PBS pH 7.4) (for vancomycin and rifampicin analysis) or 10 mL of water (for silver analysis due to the interference from sodium and phosphate ions in the ICP machine) and incubated at 37 °C for specified times. At these times, all 10 mL of buffer or water was removed and stored frozen for drug analysis. In total, 10 mL of fresh PBS or water was then replaced on top of the films, and the experiment continued. N = 4 for all release experiments. 

### 2.2. Drug Analysis 

Rifampicin was quantitated using a UV/VIS spectrometer (Cary 50, Agilent, Santa Clara, CA, USA) at 475 nm. The calibration curve was linear in the 3–50 µg/mL range and had a correlation coefficient of 0.999. Vancomycin was quantitated using HPLC methods (Waters Acquity HPLC system with empower version 1.0 software) using a mobile phase of 90% KH_2_PO_4_ (10 mM adjusted to pH 2.5) to 10% acetonitrile. This gave a peak retention time of 4 min and linear calibration in the 6–50 µg/mL range with a correlation coefficient of 0.998. Silver was analyzed using inductively coupled plasma methods (Agilent ICP-OES, Santa Clara, CA, USA). This machine gave a linear calibration in the 0.05–2 µg/mL range with a correlation coefficient of 0.99. 

## 3. Results

### 3.1. Nanofiber Characteristics

The electrospinning of PMMA nanofibers was problem-free using PEG 1000 (10% and 20%) or PEG (200 kDa) (30%), all with 3% of the three drugs. These drugs were all loaded in the single polymer nanofibers simultaneously. This produced thin (approx. 0.1 mm), fully flexible (no cracking) films. The films with 10% PEG 1000 were partially resistant to wetting but were approximately 50% soaked at 2 weeks. Films with 20% PEG 1000 were wetted moderately well over a day, and the 30% PEG 200,000-loaded PMMA films were wetted immediately. The 20% PEG-blended PMMA films had mean fiber diameters of 0.97 µm with a standard deviation of 0.39 µm (measured using a high-resolution Olympus CX410 100 X (Olympus Corporation, Hachioji, Tokyo, Japan) oil immersion lens and Olympus Stream version 1 image analysis software). There were also some stray larger diameter fibers but no areas of fibers connecting into sheets. For PLGA, it was not possible to spin (spitting) good quality films with 10% 1000 (spotty, thin, and irregular surface morphology), but films formed well with 20% PEG 1000 or 30% PEG 200 kDa, albeit with some areas of nanofiber agglomeration (this gave small areas where fibers blended into each other as the solvent dried). However, these 0.1 mm films were fully flexible and wetted immediately. Although the PMMA nanofibers were electrospun more uniformly and might be very useful for orthopedic uses, the PLGA films still formed thin sheets of drug-loaded films, which may have uses in clinical settings where biodegradation is preferred.

### 3.2. Drug Release

**Figure 1:**
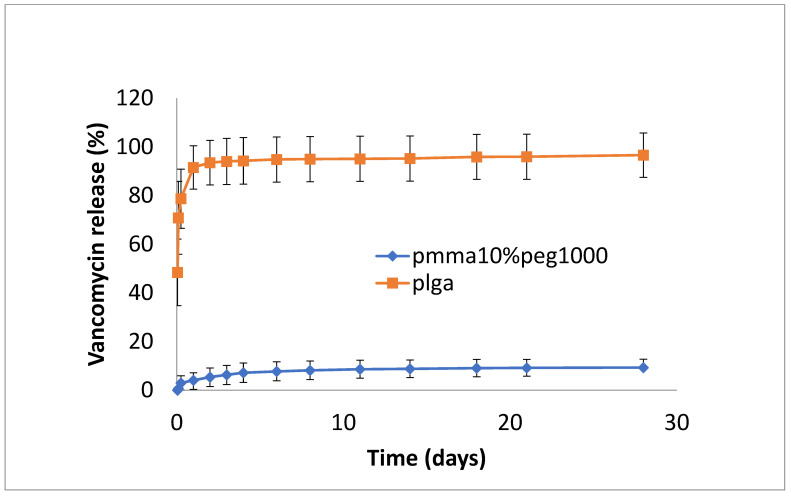
Release of vancomycin from PMMA (10% PEG 1000) or PLGA nanofiber mats.

**Figure 2:**
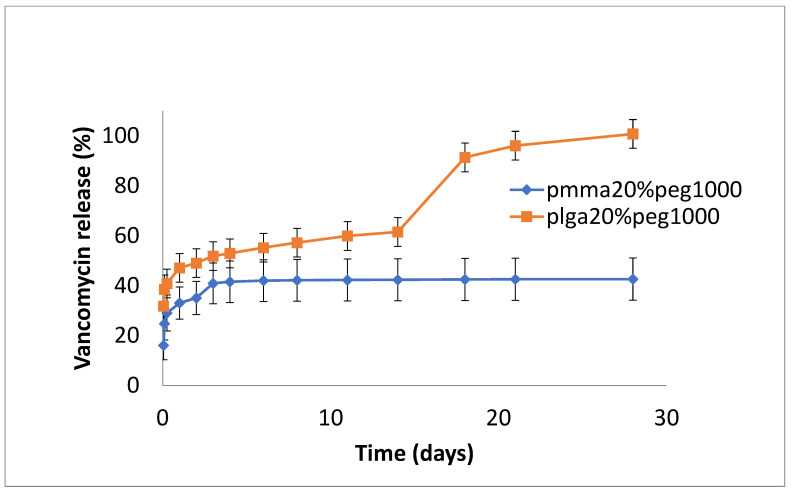
Release of vancomycin from PMMA (20% PEG 1000) or PLGA (20% PEG 1000) nanofibers.

**Figure 3:**
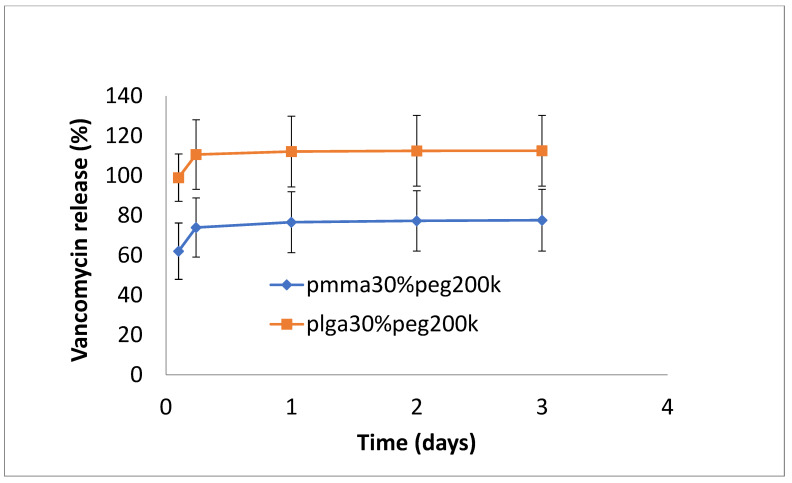
Release of vancomycin from PMMA (30% PEG 200 kDa) or PLGA (30% PEG 200 kDa) nanofibers.

The release of vancomycin from various films is shown in Figure 1, Figure 2 and Figure 3. Its release from the poorly structured films of PLGA alone was over 90% complete by day 1, with little release after that. The release from PMMA with 10% PEG1000 was slow (6% by day 3 and 9% by day 18) (Figure 1). For 20% PEG1000-blended PLGA and PMMA, film release was similar over the first 2 weeks with a 4-day burst of drug release (40–50%) flowed by slow, sustained release for PLGA (62% by day 14) but little further release for PMMA (42%) at 14 days. PLGA release then took a sharp increase so that 100% of the drug was released after 28 days (Figure 2). For 30% PEG 200 kDa-loaded films, both PLGA and PMMA showed a huge burst phase of release at 6 h (100% for PLGA and 74% for PMMA) with little release after that (Figure 3).

**Figure 4:**
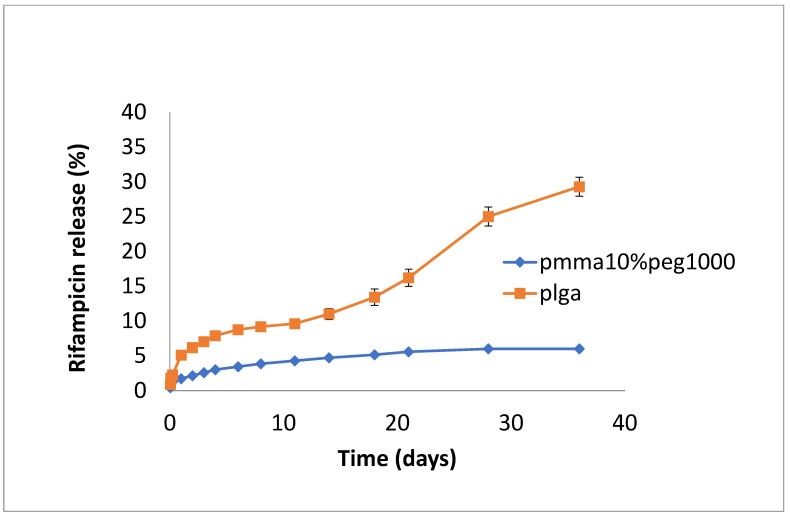
Release of rifampicin from PMMA (10% PEG 1000) or PLGA nanofibers.

**Figure 5:**
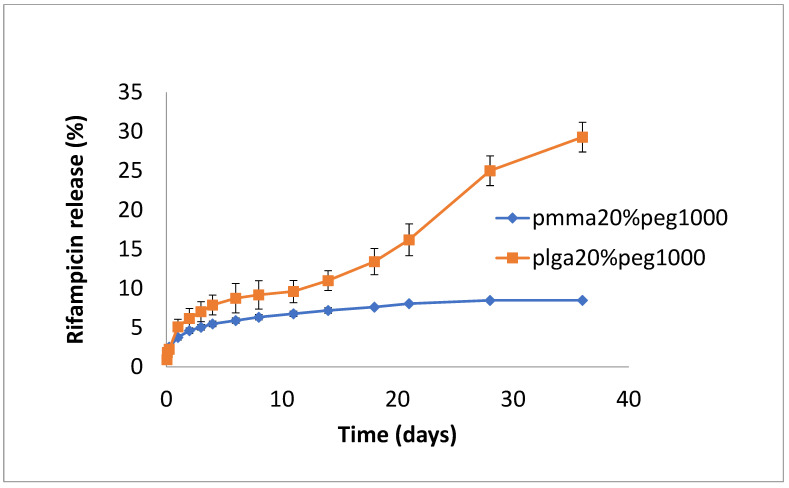
Release of rifampicin from PMMA (20% PEG 1000) or PLGA (20% PEG 1000) nanofibers.

**Figure 6:**
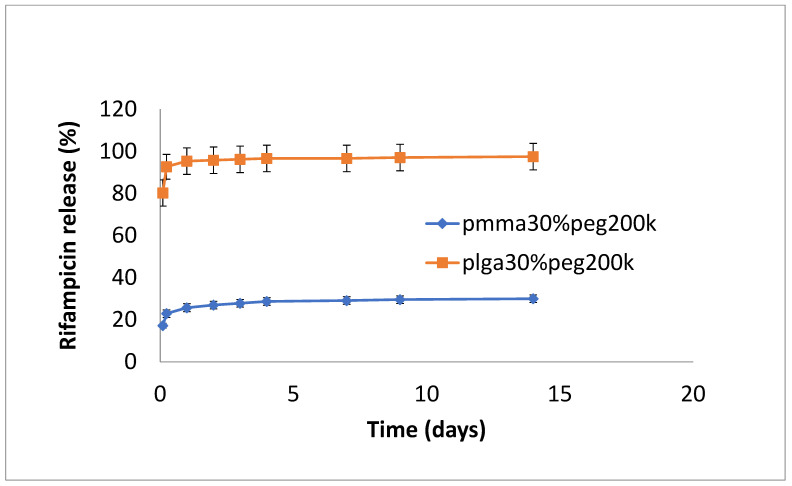
Release of rifampicin from PMMA (30% PEG 200 kDa) or PLGA (30%PEG 200 kDa) nanofibers.

The release of rifampicin showed similar profiles to vancomycin but with lower levels of drug release (Figure 4, Figure 5 and Figure 6). PLGA alone showed a steady, slow drug release with 8% released on day 4, which progressed with slow and steady release until day 14 before a sharp increase in release (29% at day 36). For PMMA, there was slow, steady release until day 21 (6%), with little release after that. (Figure 4). Films loaded with 20% PEG1000 also showed identical release profiles to the data in Figure 4, with levels of rifampicin release from PMMA a little higher at 8.5% (Figure 5). Using 30% PEG 200,000 provided almost full and immediate drug release for PLGA (95% on day 1) and increased drug release (compared to 20% PEG-loaded films) with 25% drug release on day 1, rising to 30% on day 14 from PMMA nanofibers (Figure 6). 

**Figure 7:**
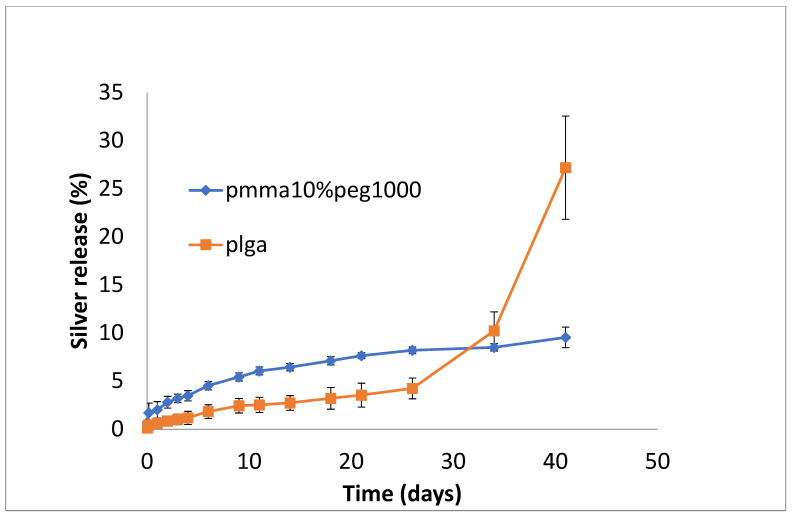
Release of silver from PMMA (10% PEG 1000) or PLGA nanofibers.

**Figure 8:**
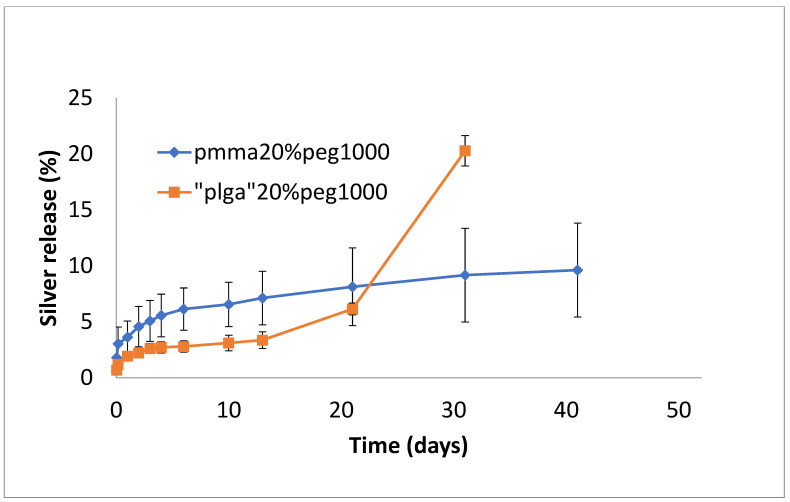
Release of silver from PMMA (20% PEG 1000) or PLGA (20% PEG 1000) nanofibers.

**Figure 9:**
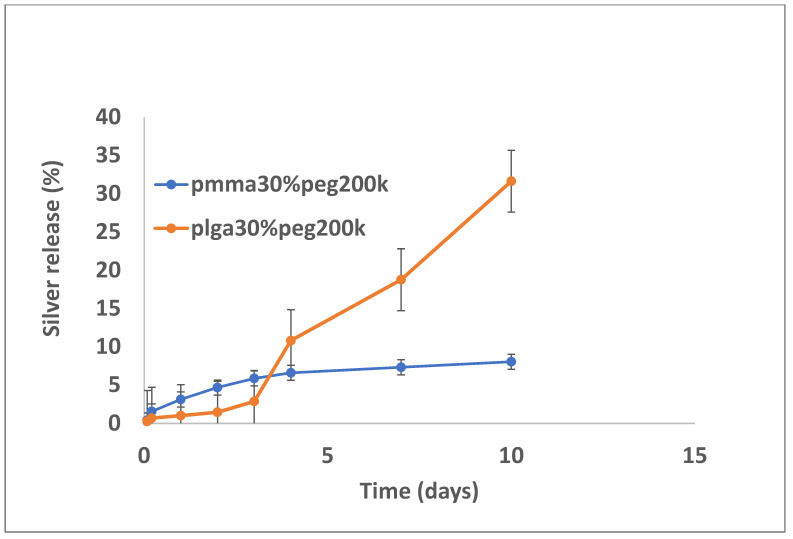
Release of silver from PMMA (30% PEG 200 kDa) or PLGA (30% PEG 200 kDa) nanofibers.

Silver release from both PLGA and PMMA was slow and steady for all formulations (Figure 7, Figure 8 and Figure 9). In total, 6–8% was released at 10 days, irrespective of PEG loading. For PLGA, lower initial release rates (e.g., 1–3% on day 3) were followed by a slow steady release. For no PEG and 20% PEG1000 samples, PLGA rates increased rapidly after day 20 to levels of 25%, and for 30% drug PEG-loaded samples, release rates increased earlier after 4 days.

## 4. Discussion

For use in orthopedic settings that require bone cement, drug-loaded, controlled-release polymeric formulations should also be manufactured from the same material (PMMA). For other surgeries, the further choice of a degradable (PLGA) formulation might also be preferred. In this study, both PMMA and PLGA electrospun nanofiber mats containing a triple antibiotic payload were successfully manufactured to provide some level of controlled release of all agents. 

Rifampicin is especially active against *S. Aureus* (including MRSA) and especially good at penetrating biofilms [7,18], which indicates it to be an appropriate antibiotic for use in orthopedic settings. However, because resistance to this drug can occur very rapidly, it is usually used in combination with other antibiotics, including vancomycin, in orthopedic settings [4,6,7]. This combination is very successful against both Gram-positive and -negative bacteria [2,6]. Aragon et al. encapsulated rifampicin in PLGA microspheres for decorating polycaprolactone (biodegradable) nanofibers [16]. This formulation released 60% of the drug by day 1 and 80% by day 28 and achieved good antibacterial effects against both Gram-positive (at 20 ppm) and Gram-negative bacteria (at 90 ppm). The use of PLGA nanofibers to encapsulate and release vancomycin and ceftazidime has been previously described [19]. Following wrapping around a stainless-steel implant, these formulations were demonstrated to provide high local concentrations in vivo for effective bacterial killing [19]. Similarly, Chen et al. [20] encapsulated vancomycin and gentamicin in PLGA nanofiber mats to provide the antibacterial concentrations of drugs for 3 weeks. 

Silver has been shown to greatly increase the antibiotic effect of vancomycin against both Gram-positive and Gram-negative bacteria [11,12], and Varisco et al. [13] showed that when silver–vancomycin combinations are coated on implants, there were good antibacterial effects against *S. Aureus*. Similarly, the addition of silver to rifampicin formulations improved the antibiotic effect against MRSA biofilms [14] and generally improved the antibiotic spectrum of rifampicin [15]. 

In this study, the hydrophobic sulfadiazine form of silver (used in commercial topical antibiotic formulations), which is soluble in organic solvents, was used in the electrospinning process along with vancomycin and rifampicin. Using 3% (*w*/*w* drug to polymer) loadings, all three drugs were encapsulated effectively in both PMMA and PLGA nanofiber mats. Zaszczynska et al. [21] electrospun PMMA-HAP nanofibers for orthopedic use demonstrated good osteoblast attachment and proliferation (which is good for binding), but water contact angles were still unfavorably high (130 degrees). Similarly, Wang et al. [22] reported high contact angles for poly lactic acid nanofibers. We previously showed that the addition of PEG1000 to PMMA nanofibers significantly reduces the contact angle, improving wettability and increasing the release of the antifibrotic drug Kynurenic acid [17]. Similarly, in this study, the addition of PEG1000 at 20% *w*/*w* rendered the nanofiber mats wettable (which is important in clinical settings) and provided the controlled release of all drugs (Figure 2, Figure 5 and Figure 8). Because all these drugs are effective at low microgram/mL levels, the local concentrations of these agents are likely to vastly exceed the required bactericidal concentrations for extended periods. The additive or synergistic activity of silver release may further improve the antibacterial effects, especially against drug-resistant bacteria, where higher drug concentrations are needed. In order to electrospin nanofibers with a higher PEG content, the higher molecular weight form (200 kDa) of PEG with a much higher melting point than PEG1000 was used. Despite forming good-quality nanofiber mats, the drug release rates were generally too high for extended use in vivo (Figure 3, Figure 6 and Figure 9). 

## 5. Conclusions

In this study, a combination of three antibiotic drugs was simultaneously loaded into PMMA or PLGA nanofibers and provided some level of controlled release for each drug. These combinations of drugs may provide a super potent regime for killing Gram-positive and -negative bacteria, especially drug-resistant species encountered in orthopedic settings. The addition of PEG allowed a reduction in the (unwanted) hydrophobic nature of the nanofibers so that rapid wetting could occur in vivo. The nanofibers mats are thin, flexible, and easy to wrap around implants and can fit any cavity or surface. Further studies hope to test the in vivo antibacterial effect of both triple drug-loaded PMMA and PLGA nanofiber mats.

## Data Availability

Data is available to readers upon request.

## References

[1] Mancino F., Gant V., Meek D.R.M., Haddad F.S. (2023). Vancomycin powder in total joint replacement. Bone Jt. J..

[2] Hipfl C., Leopold V., Becker L., Pumberger M., Perka C., Hardt S. (2023). Two-stage revision for periprosthetic joint infection in cemented total hip arthroplasty: An increased risk for failure?. Arch. Orthop. Trauma Surg..

[3] Dedeogullari E.S., Caglar O., Danisman M., Tokgozoglu A.M., Kamaci S., Atilla B. (2023). Low dose vancomycin-loaded spacers for two-stage revision knee arthroplasty: High success, low toxicity. Knee.

[4] Corro S., Vicente M., Rodriguez-Pardo D., Pigrau C., Lung M., Corona P.S. (2020). Vancomycin-Gentamicin Prefabricated Spacers in 2-Stage Revision Arthroplasty for Chronic Hip and Knee Periprosthetic Joint Infection: Insights into Reimplantation Microbiology and Outcomes. J. Arthroplast..

[5] Xie L., Zhu J., Yang M., Yang C., Luo S., Xie Y., Pu D. (2017). Effect of Intra-wound Vancomycin for Spinal Surgery: A Systematic Review and Meta-analysis. Orthop. Surg..

[6] Samuel J.R., Gould F.K. (2010). Prosthetic joint infections: Single versus combination therapy. J. Antimicrob. Chemother..

[7] Rose W.E., Poppens P.T. (2009). Impact of biofilm on the in vitro activity of vancomycin alone and in combination with tigecycline and rifampicin against *Staphylococcus aureus*. J. Antimicrob. Chemother..

[8] Jackson J., Lo J., Hsu E., Burt H.M., Shademani A., Lange D. (2021). The Combined Use of Gentamicin and Silver Nitrate in Bone Cement for a Synergistic and Extended Antibiotic Action against Gram-Positive and Gram-Negative Bacteria. Materials.

[9] Jackson J., Leung F., Duncan C., Mugabe C., Burt H. (2011). The use of bone cement for the localized, controlled release of the antibiotics vancomycin, linezolid, or fusidic acid: Effect of additives on drug release rates and mechanical strength. Drug Deliv. Transl. Res..

[10] Morones-Ramirez J.R., Winkler J.A., Spina C.S., Collins J.J. (2013). Silver enhances antibiotic activity against gram-negative bacteria. Sci. Transl. Med..

[11] Ni C., Zhong Y., Wu W., Song Y., Makvandi P., Yu C., Song H. (2022). Co-Delivery of Nano-Silver and Vancomycin via Silica Nanopollens for Enhanced Antibacterial Functions. Antibiotics.

[12] Hashimoto A., Miyamoto H., Kobatake T., Nakashima T., Shobuike T., Ueno M., Murakami T., Noda I., Sonohata M., Mawatari M. (2020). The combination of silver-containing hydroxyapatite coating and vancomycin has a synergistic antibacterial effect on methicillin-resistant *Staphylococcus aureus* biofilm formation. Bone Jt. Res..

[13] Varisco M., Khanna N., Brunetto P.S., Fromm K.M. (2014). New antimicrobial and biocompatible implant coating with synergic silver-vancomycin conjugate action. ChemMedChem.

[14] Farooq U., Ahmad T., Khan A., Sarwar R., Shafiq J., Raza Y., Ahmed A., Ullah S., Rehman N.U., Al-Harrasi A. (2019). Rifampicin conjugated silver nanoparticles: A new arena for development of antibiofilm potential against methicillin resistant *Staphylococcus aureus* and *Klebsiella pneumoniae*. Int. J. Nanomed..

[15] Ivashchenko O., Coy E., Peplinska B., Jarek M., Lewandowski M., Załęski K., Warowicka A., Wozniak A., Babutina T., Jurga-Stopa J. (2017). Influence of silver content on rifampicin adsorptivity for magnetite/Ag/rifampicin nanoparticles. Nanotechnology.

[16] Aragon J., Feoli S., Irusta S., Mendoza G. (2019). Composite scaffold obtained by electro-hydrodynamic technique for infection prevention and treatment in bone repair. Int. J. Pharm..

[17] Poormasjedi-Meibod M., Pakyari M., Jackson J.K., Elizei S.S., Ghahary A. (2016). Development of a nanofibrous wound dressing with an antifibrogenic properties in vitro and in vivo model. J. Biomed. Mater. Res. Part A.

[18] Krizsan G., Sallai I., Veres D.S., Prinz G., Szeker D., Skaliczki G. (2022). Rifampicin resistance and risk factors associated with significantly lower recovery rates after two-stage revision in patients with prosthetic joint infection. J. Glob. Antimicrob. Resist..

[19] Hsu Y.H., Chen D.W., Tai C.D., Chou Y.C., Liu S.J., Ueng S.W., Chan E.C. (2014). Biodegradable drug-eluting nanofiber-enveloped implants for sustained release of high bactericidal concentrations of vancomycin and ceftazidime: In vitro and in vivo studies. Int. J. Nanomed..

[20] Chen D.W., Liao J.Y., Liu S.J., Chan E.C. (2012). Novel biodegradable sandwich-structured nanofibrous drug-eluting membranes for repair of infected wounds: An in vitro and in vivo study. Int. J. Nanomed..

[21] Zaszczyńska A., Kołbuk D., Gradys A., Sajkiewicz P. (2024). Development of Poly(methyl methacrylate)/nano-hydroxyapatite (PMMA/nHA) Nanofibers for Tissue Engineering Regeneration Using an Electrospinning Technique. Polymers.

[22] Wang Y., Xu Y., Zhai W., Zhang Z., Liu Y., Cheng S., Zhang H. (2022). In-situ growth of robust superlubricated nano-skin on electrospun nanofibers for post-operative adhesion prevention. Nat. Commun..

